# Altered expression of A20 gene in peripheral blood mononuclear cells is associated with the progression of chronic hepatitis B virus infection

**DOI:** 10.18632/oncotarget.11993

**Published:** 2016-09-13

**Authors:** Yu-Chen Fan, Yuan-Yuan Zhang, Yan-Yan Sun, Na Wang, Xiao-Yan Xiao, Kai Wang

**Affiliations:** ^1^ Department of Hepatology, Qilu Hospital of Shandong University, Jinan 250012, China; ^2^ Institute of Hepatology, Shandong University, Jinan 250012, China; ^3^ Department of Neurology, Jinan Central Hospital affiliated to Shandong University, Jinan 250014, China; ^4^ Department of Nephrology, Qilu Hospital of Shandong University, Jinan 250012, China

**Keywords:** A20, hepatitis B virus, chronic hepatitis B, liver cirrhosis, hepatocellular carcinoma

## Abstract

A20 is an important negative immune regulator but its role in chronic hepatitis B virus (HBV) infection is still unknown. This present study was to investigate the potential role of A20 gene in the progression of chronic HBV infection. A total of 236 chronic HBV patients were included and consisted of 63 hepatocellular carcinoma (HCC), 87 liver cirrhosis (LC) and 86 chronic hepatitis B (CHB). The mRNA level of A20 gene in peripheral blood mononuclear cells was determined using quantitative real-time polymerase chain reaction. Receptor operating characteristic curve (ROC) was performed to determine the diagnostic value of A20 mRNA in different stages of chronic HBV infection. A20 mRNA levels in all HBV patients were significantly higher than healthy controls (n=30), of whom HCC and LC patients showed higher A20 mRNA level than CHB patients. In CHB patients, A20 mRNA was closely associated with alanine aminotransferase (ALT), aspartate aminotransferase (AST) and total bilirubin. In LC patients, A20 mRNA was significantly associated with ALT, AST, albumin, haemoglobin and platelet. In HCC patients, elevated A20mRNA was also observed in patients with vascular invasion, liver cirrhosis and ascites, compared with those without. ROC analysis revealed that A20 mRNA could effectively discriminate LC from CHB, decompensated LC from compensated LC, and HCC from CHB. In conclusion, A20 mRNA expression in peripheral blood mononuclear cells was associated with dynamic progression of chronic HBV infection. A20 gene might be a potential biomarker to determine the different stages of chronic HBV infection.

## INTRODUCTION

Hepatitis B virus (HBV) infection is a serious and prevalent public health problem, with more than 350 million subjects are chronic HBV carriers in the world [1]. Chronic HBV infection is at a lifelong risk for the development of chronic hepatitis B(CHB), liver cirrhosis(LC), and hepatocellular carcinoma(HCC) [2]. About 10% of compensated liver cirrhosis will develop into decompensated cirrhosis each year, and once the decompensation occurred, the mortality will be raised as high as 85% over 5 years [3]. The progression of liver pathology, ranging from chronic inflammation and fibrosis, cirrhosis, even to carcinogenesis, have been demonstrated to be exactly orchestrated by the complex interaction between HBV and host immune response [4]. Understanding the dynamic progression of chronic HBV infection is important for decreasing the incidence of HBV associated decompensated complications including LC and HCC. Aberrant immune response might contribute to the progression of chronic HBV infection. However, the precise mechanism underlying the natural course of HBV infection is still unknown.

A20, also known as tumor necrosis factor α-induced protein 3, was recently identified as a primary response gene stimulated by the treatment of tumor necrosis factor alpha in endothelial cells and thereafter was suggested to be an important immune negative regulator in inflammation and immunity [5–7]. As an inhibitor of nuclear factor-κB (NF-κB), A20 can restrict cellular signal pathway transduced from tumor necrosis factor receptors, toll-like receptors, nucleotide-binding oligomerization domain containing 2 receptors or T cell receptors [8, 9]. A20-deficient mice would display severe inflammation and tissue damage in multiple organs and eventually develop into death [10]. In addition, A20 also functions to restrict innate antiviral signaling responses via the regulation of interferon regulatory factor and NF-κB pathway induced by virus infection [11]. Besides, A20 has the capacity to suppress T cell activation and limit T cell receptor signaling to NF-κB by cleaving Malt1 ubiquitin chains [12]. A20-silenced dendritic cells showed the unique abilities to inhibit regulatory T cells and activate cytotoxic T lymphocytes and T-helper cells [13]. Accelerating evidences suggested that A20 might play an important role in the development of chronic inflammatory diseases, autoimmune disorders, B cell lymphomas and tumors [14–20]. In carcinogenesis of HCC, A20 has been reported to play a negative role in the development and progression of HCC probably through inhibiting Twist1 expression [21, 22]. Another report showed that hepatocyte-specific A20 knockout mice displayed the characteristics of chronic liver inflammation and showed increased susceptibility to chemically or high fat-diet-induced hepatocellular carcinoma development [23]. And they therefore concluded that A20 might prevent chronic liver inflammation and carcinogensis by protecting hepatocytes from death [23]. Additionally, the role of A20 has been demonstrated in hepatitis C infection, where the down-regulated expression of A20 gene is correlated with interferon-α-based antiviral therapy [24]. Our group recently have reported the levels of A20 mRNA in the immune clearance phase and hepatitis B negative phase were significantly higher than that in immune tolerance phase and low-replicative phase [25]. And we also demonstrated that the up-regulation of the A20 gene might contribute to the severity of HBV associated liver failure using internal and validation cohort [26].However, there was still no clinical data for the dynamic expression of A20 gene in different stages of chronic HBV infection ranging from CHB, LC to HCC.

In this study, we performed quantitative real time-polymerase chain reaction (RT-qPCR) to determine the mRNA expression of A20 in peripheral blood mononuclear cells (PBMCs) from a cross sectional cohort and to explore the potential associations of A20 gene with clinical parameters in different stages of chronic HBV infection.

## RESULTS

### The characteristics of all the subjects included in the study

**Figure 1:**
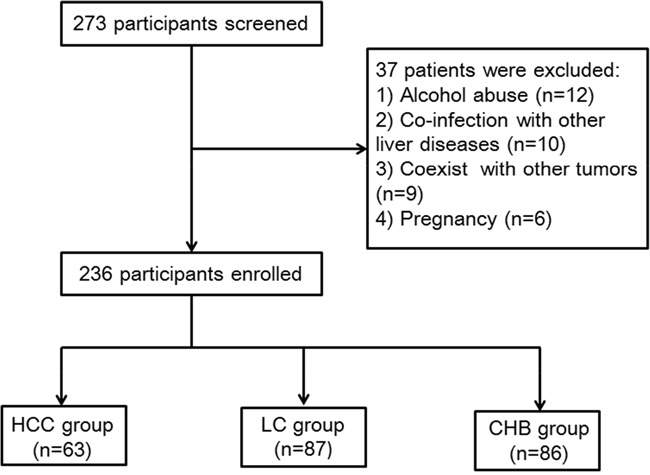
Flowchart for the selected procession of all the patients in this present study

Figure 1 displayed the flowchart for the inclusion of all the patients in this cross sectional study. From September 2013 to November 2014, a total of 273 patients with HBsAg positive were screened at the department of Hepatology, Qilu Hospital of Shandong University. Of whom, a total of 37 patients were excluded as the following reasons: 12 for severe alcohol abuse, 10 for co-infection with other liver diseases, 9 for coexist with other tumors, 6 for pregnancy. Finally, a total of 236 patients were enrolled in this study. In addition, 30 healthy volunteers were collected as controls. The basic characteristic of all the subjects were presented in Table 1.

**Table 1:** The basic characteristic of the enrolled subjects

Variable	HCC (n=63)	LC (n=87)	CHB (n=86)	HCs (n=30)	*P*-value
Male (%)	47 (74.6)	65 (74.7)	49 (72.1)	16 (53.3)	0.133^a^
Age (years)	59 (52-62)	58 (50-63)	59 (53-64)	55 (45-61)	0.160^b^
HBsAg (IU/mL)	4887 (134-6598)	5175 (972-6819)	2157 (838-5661)	NA	0.252^b^
HBeAg+(%)	22 (34.9)	58 (66.7)	50 (73.5)	NA	<0.001^a^
HBV DNA+(%)	27 (42.9)	61 (70.1)	54 (79.4)	NA	<0.001^a^
WBC (10^9^/L)	6.01 (4.72-8.23)	5.23 (3.43-6.98)	5.35 (4.52-6.23)	NA	0.050^b^
HGB (g/L)	134 (118-145)	132 (114-141)	147 (138-153)	NA	<0.001^b^
PLT (10^9^/L)	149 (100-243)	103 (82-145)	197 (156-230)	NA	<0.001^b^
ALT (U/L)	56 (38-139)	52 (35-93)	120 (51-263)	NA	<0.001^b^
AST (U/L)	57 (40-88)	54 (31-70)	55 (35-123)	NA	0.181^b^
ALB (g/L)	35.9 (32.8-39.9)	32.7 (29.4-38.8)	41.4 (37.7-44.8)	NA	<0.001^b^
TBIL (umol/L)	33.1 (16.5-52.2)	27.9 (17-58)	38.1 (16.4-94.7)	NA	0.754^b^
PTA (%)	74 (62-82)	70 (55-81)	97 (90-105)	NA	<0.001^b^
PT-INR	1.13 (1.07-1.24)	1.17 (1.03-1.34)	0.99 (0.94-1.04)	NA	<0.001^b^
Cr (umol/L)	67 (57-74)	63 (55-80)	60 (50-68)	NA	0.010^b^
MELD score	6.52 (5.07-9.30)	6.99 (4.32-11.65)	NA	NA	0.702^c^
AFP (ng/mL)	70.8 (15.3-242)	14.2 (4.2-38.1)	10.5 (5.0-34.7)	NA	<0.001^b^
Ascites (%)	21 (33.3)	33 (37.9)	NA	NA	0.267^a^
HE (%)	13 (20.6)	12 (13.8)	NA	NA	0.563^a^
Variceal bleeding (%)	28 (44.4)	23 (26.4)	NA	NA	0.022^a^

Quantitative variables were expressed as the median (centile 25; centile 75).

Categorical variables were expressed as number (%).

a Chi-square test;

b Kruskal-Wallis test;

c Mann-Whitney U test.

HCC, hepatocellular carcinoma; LC, liver cirrhosis; CHB, chronic hepatitis B; HCs, healthy controls; HBsAg, hepatitis B surface antigen; HBeAg, hepatitis B e antigen; WBC, white blood cell; HGB, haemoglobin; PLT, platelet; ALT, alanine aminotransferase; AST, aspartate aminotransferase; ALB, albumin; TBIL, total bilirubin; PTA, prothrombin time activity; PT-INR, prothrombin time-international normalized ratio; Cr, creatinine; MELD = model for end-stage liver disease; AFP, alpha-fetoprotein; HE = hepatic encephalopathy; NA, not available.

### Expression of A20 mRNA from PBMCs in different stages of chronic HBV infection patients and healthy controls

**Figure 2:**
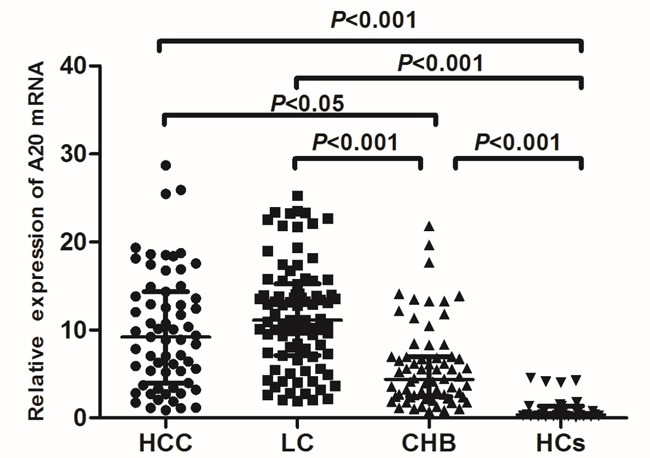
The comparison of A20 mRNA level among different progression of hepatitis B infection The significance of differences was calculated using the Kruskal-Wallis test.

In this study, we first determined the expression of A20 mRNA in PBMCs using quantitative RT-PCR from all the patients with chronic HBV infection and healthy controls. The relative expression of A20 mRNA in patients with HCC, LC, CHB was significantly higher than that in healthy controls (median (centile 25; centile 75) HCC: 9.20 [3.97-14.36]; LC: 11.10 [7.10-15.23]; CHB: 4.35 [2.42-6.96]; HCs: 0.35 [0.22-1.36], *P*<0.001, respectively) (Figure 2). In addition, the relative expression of A20 mRNA in patients with HCC, LC was significantly higher than that in CHB patients (HCC vs CHB: 9.20 [3.97-14.36] vs 4.35 [2.42-6.96], *P*<0.05; LC vs CHB: 11.10 [7.10-15.23] vs 4.35 [2.42-6.96], *P*<0.001) (Figure 2). These data suggested that A20 might participate in the progression of chronic HBV infection.

### Associations between A20 mRNA level and clinical features in patients with chronic hepatitis B

**Figure 3:**
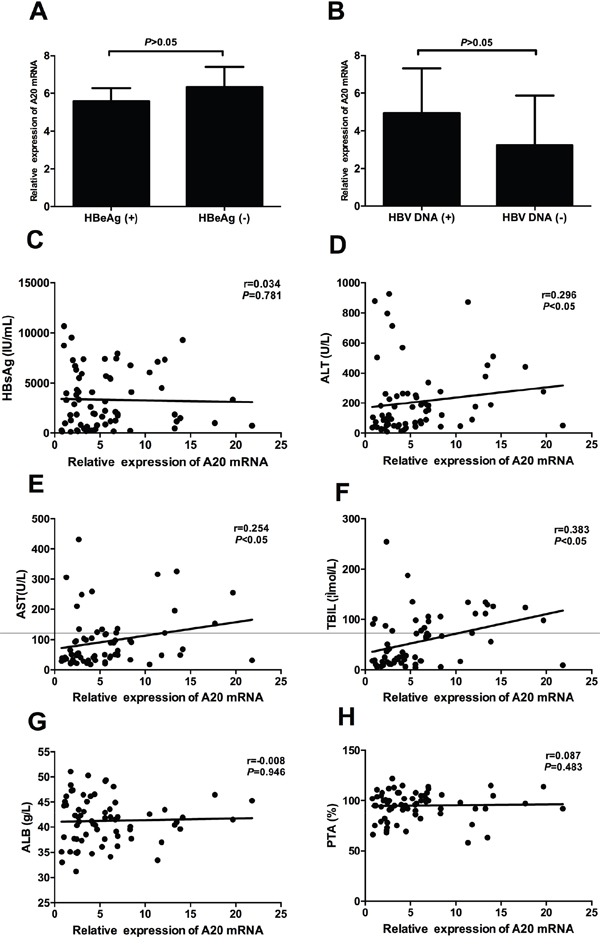
Correlation between relative expression of A20 mRNA and clinical parameters of patients with chronic hepatitis B **A.** No significant difference was found between HBeAg (+) group and HBeAg (-) group; **B.** No significant difference was found between HBV DNA (+) group and HBV DNA (-) group; **C.** No significant correlation was found between A20 mRNA and HBsAg; **D-F.** Significant correlations were found between A20 mRNA and ALT, AST and TBIL; **G-H.** No significant correlations were found between A20 mRNA and ALB and PTA.

Chronic hepatitis B accounts for almost 80% of chronic HBV infection and is a primary disease condition that usually tend to liver cirrhosis and liver cancer without effective treatment. Therefore, we evaluated the possible correlations between A20 mRNA and clinical parameters in CHB patients. There were no significant differences of A20 gene in the HBeAg positive and negative CHB patients, and HBV DNA positive and negative CHB patients (Figure 3A and 3B). Furthermore, we demonstrated that the relative expression of A20 mRNA was significantly positively correlated with alanine aminotransferase (ALT) (r=0.296, *P*<0.05), aspartate aminotransferase (AST) (r=0.254, *P*<0.05) and total bilirubin (TBIL) (r=0.383, *P*<0.05) (Figure 3D, 3E and 3F). However, there were no significant correlations between A20 gene and albumin (ALB) (r=-0.008, *P*=0.946), prothrombin time activity (PTA) (r=0.087, *P*=0.483) or HBsAg titer (r=0.034, *P*=0.781) (Figure 3C, 3G and 3H).

### Associations between A20 mRNA level and clinical parameters in patients with liver cirrhosis

**Figure 4:**
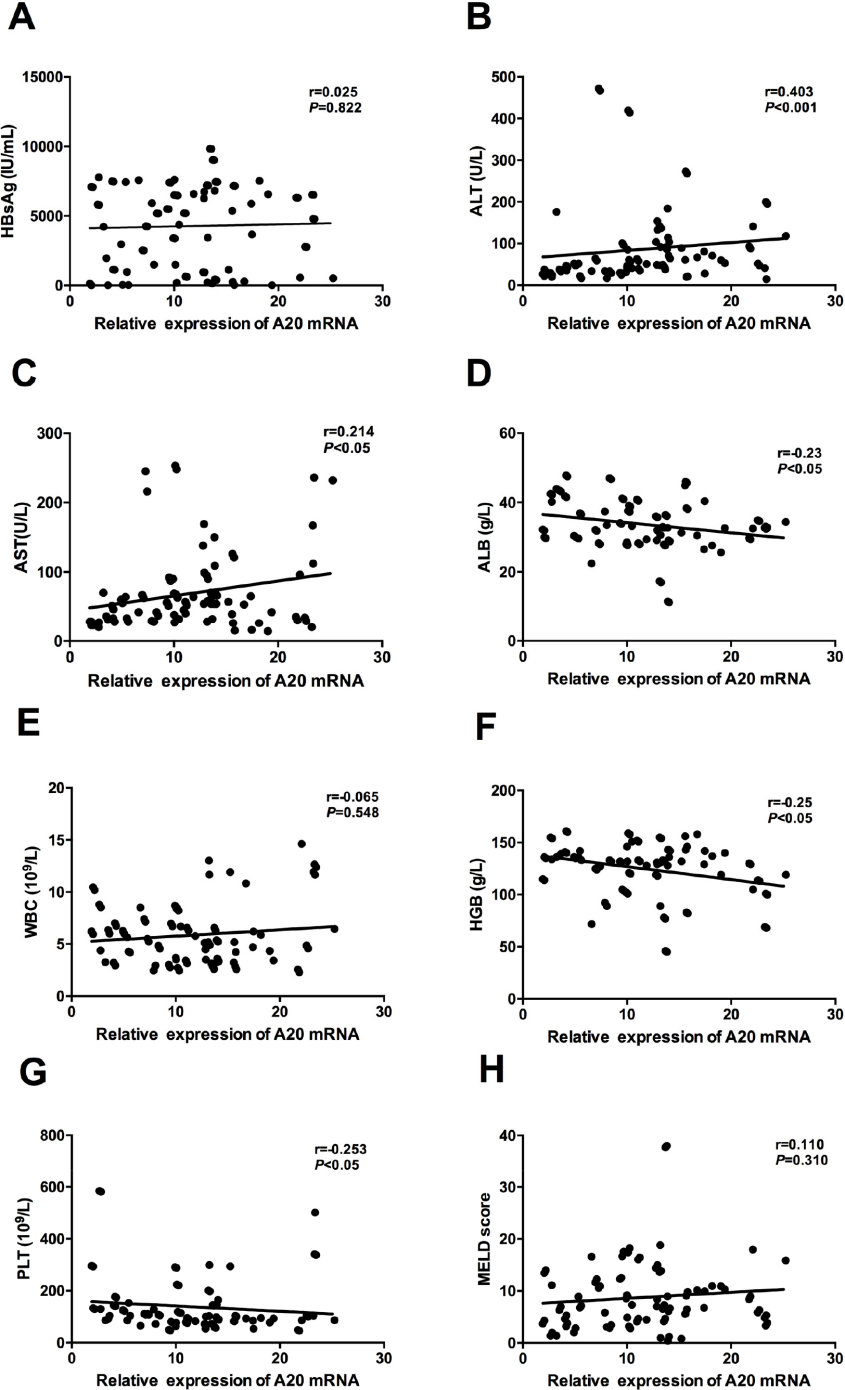
Correlation between relative expression of A20 mRNA and clinical parameters of patients with liver cirrhosis **A.** No significant correlation was found between A20 mRNA and HBsAg; **B-D.** Significant correlations were found between A20 mRNA and ALT, AST and ALB; **E.** No significant correlation was found between A20 mRNA and WBC; **F-G.** Significant correlations were found between A20 mRNA and HGB and PLT; **H.** No significant correlation was found between A20 mRNA and MELD score.

**Figure 5:**
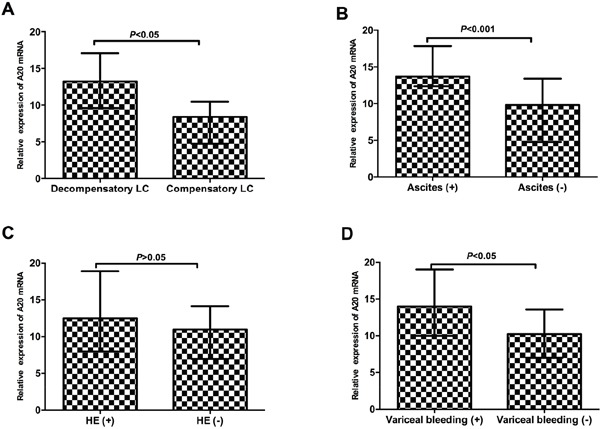
The correlations between A20 mRNA and the common complications of liver cirrhosis **A.** A20 mRNA level was increased in decompensated liver cirrhosis compared with compensated liver cirrhosis; **B.** A20 mRNA level was increased in ascites group compared with non-ascites group; **C.** No significant difference was found between hepatic encephalopathy group and non- hepatic encephalopathy group; **D.** A20 mRNA level was increased in variceal bleeding group compared with non- variceal bleeding group.

**Figure 6:**
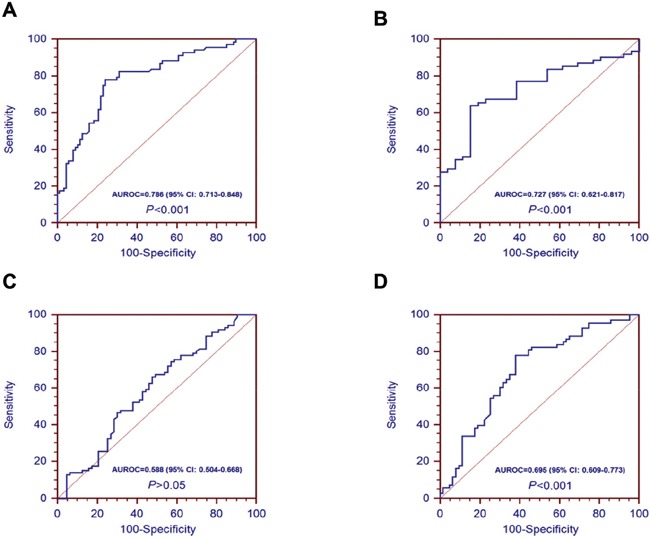
Receiver operating characteristic (ROC) curves for A20 mRNA in patients with hepatitis B infection **A.** ROC curves of A20 mRNA level in discriminating liver cirrhosis from chronic hepatitis B; **B.** ROC curves of A20 mRNA level in discriminating decompensated liver cirrhosis from compensated liver cirrhosis; **C.** ROC curves of A20 mRNA level in discriminating hepatocellular carcinoma from liver cirrhosis; **D.** ROC curves of A20 mRNA level in discriminating hepatocellular carcinoma from chronic hepatitis B.

In Figure 2, we have reported that A20 mRNA level in liver cirrhosis was significantly higher than patients with chronic hepatitis B. These results might revealed the potential role of A20 in the progression to liver cirrhosis from chronic hepatitis B. Here, we analyzed the potential relationships between A20 mRNA and clinical parameters in patients with liver cirrhosis. We demonstrated that A20 mRNA was significantly associated with ALT (r=0.403, *P*<0.001), AST (r=0.214, *P*<0.05), ALB (r=-0.23, *P*<0.05), haemoglobin (HGB) (r=-0.25, *P*<0.05) and platelet (PLT) (r=-0.253, *P*<0.05) (Figure 4B, 4C, 4D, 4F and 4G). However, there were no significant associations between A20 mRNA and HBsAg (r=0.025, *P*=0.822), white blood cell(WBC) (r=-0.065, *P*=0.548) or model for end-stage liver disease (MELD) score (r=0.110, *P*=0.310) (Figure 4A, 4E and 4H). Further, we also determined the potential role of A20 mRNA in compensated stage and decompensated stage of liver cirrhosis. Figure 5A showed that the A20 mRNA level in decompensated LC patients was significantly higher than that in compensated LC patients (13.23 [9.61-17.07] vs 8.40 [4.74-10.50], *P*< 0.05). In addition, we analyzed the correlations between A20 mRNA and the common manifestations of decompensated LC, including ascites, variceal bleeding and hepatic encephalopathy. The results demonstrated that decompensated LC patients with ascites or variceal bleeding had significantly higher A20 mRNA level than those in non-ascites or non-variceal bleeding groups (*P*<0.001, *P*<0.05, respectively) (Figure 5B and 5D). However, there was no significant differences of A20 mRNA between decompensated LC patients with hepatic encephalopathy and without hepatic encephalopathy (*P*>0.05) in Figure 5C. These results suggested the possibility that elevated expression of A20 mRNA might be involved in the pathogenesis of liver cirrhosis and closely associated with the disease severity. Furthermore, ROC analysis was performed to identify whether A20 mRNA could discriminate LC from CHB, and decompensated LC from compensated LC. Figure 6A showed the area under the receiver operating characteristic(AUROC) of A20 mRNA for the diagnosis of LC from CHB 0.786 (95% confidence interval 0.713-0.848, *P*< 0.001), and the optimal cutoff value was 6.97 with the sensitivity of 77.9%, specificity of 75.9%, positive predictive value (PPV) of 71.6%, negative predictive value (NPV) of 81.5%, positive likelihood ratio (PLR) of 3.23 and negative likelihood ratio (NLR) of 0.29. Meanwhile, the AUROC of A20 mRNA for the diagnosis of decompensated LC from compensated LC individuals was 0.727 (95% confidence interval 0.621-0.817, *P*< 0.001), and the optimal cutoff value was 11.1 with the sensitivity of 63.9%, specificity of 84.6%, PPV of 90.7%, NPV of 50.0%, PLR of 4.16 and NLR of 0.43 in Figure 6B.

### Associations between A20 mRNA level and clinical features in patients with hepatocellular carcinoma

The associations between relative expression of A20 mRNA and clinical characteristics of the HCC patients were shown in Table 2. There were no associations between relative expression of A20 mRNA and gender, age, HBeAg, HBV DNA, smoking, alcohol, serum alpha-fetoprotein (AFP) levels, hepatic encephalopathy, variceal bleeding, primary tumor number, tumor size or tumor node metastasis (TNM) staging. However, the relative expression of A20 mRNA was significantly correlated with liver cirrhosis, ascites and vascular invasion of HCC (*P*<0.05, respectively). Furthermore, ROC analysis was performed to identify whether A20 mRNA could discriminate HCC from LC patients, and HCC from CHB patients. Figure 6C showed the AUROC of A20 mRNA could not effectively discriminate HCC from LC patients(*P*>0.05). The AUROC of A20 mRNA for the diagnosis of HCC from CHB individuals was 0.695 (95% confidence interval 0.609-0.773, *P*< 0.001), and the optimal cutoff value was 6.97 with the sensitivity of 77.9%, specificity of 61.9%, PPV of 68.8%, NPV of 72.7%, PLR of 2.05 and NLR of 0.36 in Figure 6D.

**Table 2:** Correlation between A20 mRNA expression and clinicopathological parameters in HCC patients

Parameters	Total number	Relative expression of A20 mRNA	*P*-value
**Gender**			0.352
Male	47	9.84 (5.35-14.87)	
Female	16	7.42 (3.57-12.70)	
**Age (years)**			0.133
≤60	34	11.86 (3.62-16.80)	
>60	29	7.01 (4.59-10.74)	
**HBeAg**			0.261
Negative	41	6.11 (3.01-16.84)	
Positive	22	10.08 (7.11-12.99)	
**HBV DNA**			0.123
Negative	36	10.39 (5.23-17.99)	
Positive	27	7.14 (3.71-12.40)	
**Smoking**			0.361
No	25	7.04 (3.27-15.88)	
Yes	38	10.20 (5.51-13.95)	
**Alcohol**			0.890
No	28	8.34 (4.28-16.32)	
Yes	35	9.37 (3.71-13.58)	
**LC**			0.010
Negative	14	5.27 (2.01-6.29)	
Positive	49	10.35 (6.45-14.93)	
**Ascites**			0.021
Negative	42	7.09 (3.91-11.78)	
Positive	21	14.87 (5.81-18.27)	
**HE**			0.292
Negative	50	8.61 (3.84-13.64)	
Positive	13	12.40 (5.36-16.84)	
**Variceal bleeding**			0.688
Negative	35	8.85 (3.33-16.90)	
Positive	28	9.98 (5.92-12.83)	
**AFP**			0.379
≤20	22	7.03 (2.60-16.80)	
>20	41	9.84 (5.72-13.37)	
**Primary tumor number**			0.076
Single	32	7.38 (3.43-11.70)	
Multiple	31	11.70 (5.56-16.90)	
**Tumor size**			0.783
≤3cm	29	10.05 (4.46-14.62)	
>3cm	34	8.10 (3.84-14.58)	
**Venous invasion**			0.040
Negative	43	7.14 (3.33-12.40)	
Positive	20	13.26 (6.89-17.53)	
**TNM staging**			0.188
I/II	39	7.83 (3.71-12.53)	
III/IV	24	10.54 (5.74-17.53)	

HCC, hepatocellular carcinoma; HBeAg, hepatitis B e antigen; LC, liver cirrhosis; HE = hepatic encephalopathy; AFP, alpha-fetoprotein; TNM, tumor node metastasis.

## DISCUSSION

A20 is an immune negative regulatory molecule the inflammation and immunity during virus infection. Complex immune networks contribute to the progression of chronic hepatitis B virus infection. However, the expression of immune negative molecules such as A20 have not been well characterized up to date. In the present study, we first reported the dynamic expression of A20 gene in the progression of chronic HBV infection and demonstrated the correlations between A20 expression and disease progression. Our data indicated that the dynamic expression of A20 gene might involve in the progression of chronic HBV infection.

The present study demonstrated the dynamic change of A20 mRNA in different stages of chronic HBV infection. The median levels of A20 mRNA in all chronic HBV infected patients were significantly higher than healthy controls, whereas HCC and LC patients displayed increased levels of A20 mRNA than that in CHB patients. Although it was elevated at the stage of chronic HBV infection and even aggressive stages, A20 gene in PBMCs did not present continuous elevation along with disease progression from liver cirrhosis to hepatocellular carcinoma. This data suggested that A20 gene might not be the only regulator for the whole course of disease development.

As is well known, chronic hepatitis B is characterized by the persistent virus replication and liver inflammation. Thus, we first analyzed whether A20 gene is associated with serological markers for HBV infection including HBsAg, HBeAg and HBV DNA load. The presence of HBsAg is the hallmark of hepatitis B infection. HBeAg represents viral replication and the history of past infection [2]. In this present study, we did not find any correlation between A20 mRNA and serological markers for HBV replication in the patients with CHB. However, the role of A20 protein in HBV replication still needs further research. Zhang *et al.* reported that hepatitis B virus X protein could affect A20 expression at protein level but not mRNA level [27]. Next, we analyzed the correlations between A20 mRNA and liver inflammation. Serum levels of ALT and AST are the major markers for hepatic inflammation and they could partly reflect the degree of inflammatory in human liver. Serum level of TBIL is another important marker for liver injury in progressive liver diseases [28]. Our results showed that A20 gene was significantly correlated with serum makers of liver injury. Overall, the above results strongly supported the hypothesis that A20 gene could play a crucial role in the pathogenesis of chronic hepatitis B.

Cirrhosis is an advanced stage of liver fibrosis and is always characterized by the impaired liver function, increased intrahepatic resistance and high risk for the development of hepatocellular carcinoma [3]. Our present study is the first report to investigate the expression of A20 gene in liver cirrhosis during chronic HBV infection. Our results showed that A20 gene was increased in the patients from chronic hepatitis B to liver cirrhosis. Furthermore, we also demonstrated that A20 gene was closely associated with AST, ALB, HGB and PLT. Abnormal aminotransferase level is often closely associated with liver injury, while the decrease of albumin is the manifestation of liver dysfunction. These results indicated that A20 gene might contribute to liver injury in cirrhotic patients. In addition, decreased level of peripheral blood cell counts usually occur due to the strengthened splenic pathological function which resulted mainly from portal hypertension in liver cirrhosis. In this present study, we also found that A20 gene was also increased in decompensated LC patient compared with compensated LC patients. Major clinical complications of cirrhosis include ascites, renal failure, hepatic encephalopathy, and variceal bleeding [29]. We demonstrated that cirrhotic patients with ascites, variceal bleeding had significantly higher A20 mRNA than that in non- ascites or non- variceal bleeding. These results supported the hypothesis that A20 might play an important role in the severity of liver cirrhosis. Of note, we reported that A20 mRNA have significant power in discriminating LC from CHB, and decompensated LC from compensated LC. These results might provide a new diagnosis tool in early detecting LC from CHB, and decompensated LC from in compensated LC.

Hepatocellular carcinoma is the sixth most common neoplasm and the third most frequent cause of cancer death [30]. In China, the dominant risk factor for hepatocellular carcinoma is chronic HBV infection. In this study, we showed that the relative expression of A20 mRNA was significantly higher in HCC patients than that in CHB patients and HCs, which indicated that A20might exert its function as a tumor enhancer in HBV-related HCC. As a negative immune regulator, A20 may allow HBV to escape the immune response and cause immune tolerance, thereby contributing to the development of HBV-related HCC. These results were consistent with a previous research in which a strong expression of A20 protein was found in all of the liver tumor tissues compared with normal human liver tissues [31]. We have also reported that the increased expression of A20 mRNA strongly correlated with liver cirrhosis, ascites and vascular invasion of HCC. Moreover, we have also reported that the level of A20 mRNA have significant power in discriminating HCC from CHB. However, elucidating the exact mechanisms for the involvement of A20 in HCC development still requires further study.

There are still several limitations in the present study. First, A20 is a classic negative feedback regulator and its induction and regulation appears to be far more complicated. The precise mechanism for the role of A20 in chronic HBV infection are required to be further studied. Second, the sample number of this cross sectional cohort from our single institution is rather low and it is indeed our limitation in our present study. In our present study, the number of patients and healthy controls have strictly calculated using PASS software 11.0 and could reach statistical significance. However, it would have been more useful to follow patients in a longer-term, preferably in a multi-center study with a larger population.

In summary, our study demonstrated the dynamic change of A20 gene in different progression of chronic HBV infection ranging from chronic hepatitis, liver cirrhosis to hepatocellular carcinoma, and revealed A20 might contribute to the disease severity in the whole course of chronic HBV infection. Our study also indicated that early detection of A20 gene in PBMCs might be of help in the early diagnosis of progressive liver diseases during chronic HBV infection.

## PATIENTS AND METHODS

### Subjects

A total of 86 patients with CHB, 87 patients with LC, 63 patients with HCC and 30 healthy controls who were admitted to the Department of Hepatology, Qilu Hospital of Shandong University between September 2013 and November 2014 were enrolled in this study. According to the 2009 update of the American Association for the Study of Liver Diseases Practice Guidelines for Management of Chronic Hepatitis B, Chronic HBV infection was defined as the presence of positive hepatitis B surface antigen (HBsAg) for over a 6-month period prior to the beginning of this study [32]. The diagnosis of liver cirrhosis was based on the presence of signs of portal hypertension, pertinent imaging features and laboratory findings, such as hypo-albuminemia, international normalized ratio (INR) increase and low PLT count. HCC was diagnosed according to the 2010 update of the American Association for the Study of Liver Diseases Practice Guidelines for Management of HCC [33]. In addition, exclusion criteria included the history of coexist with other tumors, concomitant chronic hepatitis C or human immune deficiency virus infection, pregnancy, autoimmune liver diseases, alcoholic liver diseases, nonalcoholic fatty liver diseases, drug hepatitis, and other causes of chronic liver diseases. All participants gave written informed consents under protocols approved by the Medical Ethical Committee of Qilu Hospital of Shandong University.

### Peripheral blood mononuclear cells isolation and RNA extraction

Five milliliters of ethylene diamine tetraacetic acid- anticoagulated venous peripheral blood were obtained from each subject. Ficoll-Paque Plus (GE Healthcare, Uppsala, Sweden) density gradient centrifugation was used for PBMCs isolation. Total RNA was extracted from PBMCs using TRIzol reagent (Invitrogen, Carlsbad, CA, USA) following the standard protocol provided by the manufacturer and two micrograms of total RNA were converted into cDNAs using the RevertAid™ First Strand cDNA Synthesis Kit (Fermentas, Vilnius, Lithuania).

### Quantitative real time-polymerase chain reaction

The expression of A20 gene was detected by quantitative real-time PCR method in triplicate. β-actin was used as the endogenous control. All reactions were performed using a SYBR Green PCR mix (Takara, Toyobo, Japan) on Lightcycler 480 (Roche Diagnostics, Roche Applied Science, Mannheim, Germany) according to the following protocol: denaturation at 95 °C for 30 s, followed by 40 cycles of 95 °C for 5 s, 60 °C for 30 s and 72 °C for 30 s. Primers are described as follows: A20 forward: 5′ -CGTCCAGGTTCCAGAACACCATTC-3′, and reverse: 5′-TGCGCTGGCTCGATCTCAGTTG-3′; β-actin forward: 5′-ATGGGTCAGAAGGATTCCTATGTG-3′, and reverse 5′- CTTCATGAGGTAGTCAGTCAGGTC-3′. Gene specific amplifications were demonstrated with melting curve data and gel-migration analyses. All PCR products were determined using the comparative (2 −^⊿⊿Ct^) method.

### Clinical parameters

Hepatitis B surface antigen and hepatitis B e antigen (HBeAg) were measured by an automatic analyzer (Cobas 6000 analyzer series, Roche Diagnostics, Basel, Switzerland). The HBV viral load was determined with a PCR System (ABI 7300, Applied Biosystems, Foster City, CA, USA), with a detection sensitivity of 500 IU/ml. ALT, AST, TBIL, ALB, creatinine, WBC, HGB, PLT), PT-INR and PTA were determined using standard methods in a clinical setting. MELD score was calculated on admission according to the original formula: MELD score = 3.78×LN (bilirubin [mg/dL]) + 11.2×LN (INR) + 9.57×LN (creatinine [mg/dL]) + 6.43×(etiology: 0 if cholestatic or alcoholic, 1 otherwise) [34]. Serum AFP levels were measured by electrochemiluminescence immunoassay using an automatic analyzer (COBAS e 601, Roche Diagnostics, Mannheim, Germany). Serum AFP greater than 20 ng/ml was regarded as abnormal [35].

### Statistical analysis

To estimate the group size, a pilot study was conducted using PASS software 11.0 for Windows (NCSS statistical software, Kaysville, UT, USA). We wanted the capability to show a difference of 1.0 in the A20 gene expression among the three groups. With alpha=0.05, two-tailed and a power of 90%, group size pattern as 1:2:3:3, we need at least 22:44:66:66 patients in each group. Considering a compliance rate of 90%, we asked 266 patients (30 health controls, 63 HCC, 87 LC, 86 CHB) to participate in this study. Data were analyzed by IBM SPSS 19.0 software (SPSS Inc., Chicago, IL, USA). The Kolmogorov-Smirnov test was used to examine the type of the distribution population. Continuous variables of skewed distribution were expressed as median (centile 25; centile 75). Categorical values were presented by relative frequencies. Comparison in variables were analyzed by Mann-Whitney test or Kruskal-Wallis test. The chi-square test was applied to categorical data. Correlations analysis was carried out using Spearman's rank test. Receiver operating characteristic (ROC) curves and areas under the ROC (AUROC) curves were used to assess diagnostic accuracy. All statistical analyses were two-sided, and *P* value < 0.05 was considered statistically significant.
